# Changes in rectus femoris diameter: a cooperation-independent assessment method for ICU-acquired weakness

**DOI:** 10.3389/fnut.2026.1863611

**Published:** 2026-07-24

**Authors:** Xiaoyu Peng, Peng Xu, Zhengbin Wu, Chunyan Chen, ShiLi Zhong

**Affiliations:** 1 Department of Critical Care Medicine, Chongqing Orthopedic Hospital of Traditional Chinese Medicine, Chongqing, China; 2 Department of Rehabilitation Medicine, Daping Hospital, Army Medical University, Chongqing, China; 3 Department of Critical Care Medicine, Daping Hospital, Army Medical University, Chongqing, China; 4 Department of Critical Care Medicine, Nan’an District People’s Hospital of Chongqing City, Chongqing, China

**Keywords:** electrical stimulation, ICU-acquired weakness, muscle strength, rectus femoris, Rest-contraction transverse diameter difference (ΔRF-d), Ultrasonography

## Abstract

**Background:**

ICU-acquired weakness (ICUAW) affects 50–100% mechanically ventilated critically ill patients, prolonging ventilation and hospital stay and raising long-term mortality. Conventional assessments including MRC scale and handgrip test require alert, cooperative patients, whereas electromyography and muscle biopsy are invasive and unsuitable for repeated bedside use. Static muscle ultrasound only reflects muscle mass but cannot accurately reflect actual muscle function.

**Objective:**

This study aimed to develop a cooperation-free evaluation method combining bedside ultrasound and electrical stimulation to induce rectus femoris contraction, and verify its diagnostic performance for ICUAW.

**Methods:**

A total of 58 mechanically ventilated adult patients (aged ≥18 years, mechanical ventilation duration ≥5 days) were enrolled and divided into the ICUAW group (*n* = 32, mean MRC<4) and non-ICUAW group (*n* = 26). Under supine position, rectus femoris was stimulated (30 mA, 50 Hz, 500 μs pulse). Ultrasound captured relaxed and contraction images to alculate the rest-contraction transverse diameter difference of rectus femoris (ΔRF-d, resting diameter minus contracted diameter), change in rectus femoris cross-sectional area (ΔCSA), resting thickness and CSA. Hierarchical backward stepwise multiple linear regression was performed to identify independent predictors of the mean MRC score, with 12 pre-defined clinical covariates allocated into three blocks (4 variables per block) and the within-block variable removal threshold set at *P* > 0.10. Variance inflation factor (VIF) was calculated to test multicollinearity, and receiver operating characteristic (ROC) curves were constructed to compare diagnostic performance of indicators.

**Results:**

All static and dynamic muscle indicators were significantly lower in the ICUAW group (*n* = 32) compared with non-ICUAW group (*n* = 26). ΔRF-d and corticosteroid use independently predicted MRC scores. The AUC of ΔRF-d was 0.909 (sensitivity 81.3%, specificity 92.9%), which was numerically higher than the AUC values of APACHE II (0.844), NRS-2002 (0.828), and ΔRF-CSA (0.829), indicating its relatively good discriminative capacity.

**Conclusion:**

Ultrasound-measured rest-contraction transverse diameter difference of rectus femoris (ΔRF-d, resting diameter minus contraction diameter) induced by electrical stimulation serves as a promising cooperation-independent marker to assess muscle strength and screen ICUAW in critical care.

## 1 Introduction

Over the past two decades, advances in critical care medicine have markedly improved the survival rate of critically ill patients, yet the incidence of ICU-acquired weakness (ICUAW) among ICU survivors ranges from 50 to 100% (1, 2), with the condition persisting for months to years (3–5). ICUAW comprises critical illness myopathy (CIM), critical illness polyneuropathy (CIP), and overlapping syndromes; Persistent ICU-acquired weakness (ICUAW) after ICU discharge further increases mortality risk. A cohort study demonstrated that patients with ICUAW at discharge had a higher 1-year mortality rate than those who recovered (6). This indicates that the severity and duration of ICUAW are critical determinants of long-term survival. Beyond mortality, ICUAW impairs patients’ long-term functional status and quality of life. Among COVID-19 survivors, residual ICUAW 6 months after ICU discharge was associated with reduced functional independence and greater functional dependence (7).

Current approaches for ICUAW assessment carry prominent methodological limitations. Widely used tools, including the Medical Research Council (MRC) score and handgrip strength testing (8), as well as electromyography and nerve conduction studies, all require conscious patient cooperation—a condition frequently unmet in ICU patients under sedation, delirium or impaired mental status. Although computed tomography and magnetic resonance imaging (CT/MRI) enable precise muscular morphological evaluation, they cannot be implemented at the bedside (9, 10). Neuroelectrophysiological tests are vulnerable to interference from tissue edema and environmental artifacts (11), while muscle biopsy is invasive and unsuitable for repeated measurements. Bedside muscle ultrasound, with the strengths of non-invasiveness, repeatability and operational simplicity, has been widely adopted to evaluate muscular atrophy (12, 13). Nevertheless, static morphological measurements alone cannot fully reflect actual muscle contractile function and strength.

Drawing on ultrasonic evaluation of myocardial contractile function, the present study integrated electrical stimulation and muscle ultrasound to induce passive rectus femoris contraction and quantify transverse diameter change during contraction. We aimed to establish a cooperation-free strategy for muscular strength measurement. Lower limb muscles are affected earliest in ICUAW progression (14); Studies have confirmed that dynamic changes in the cross-sectional area (RFCSA) and thickness (TH) of the rectus femoris are highly correlated with the development of intensive care unit-acquired weakness (ICUAW). Ultrasonic measurement of these two indicators can effectively predict ICUAW. Combined with its superficial anatomical location, few artifacts under electrical stimulation and clear ultrasound images, the above characteristics collectively prove that the rectus femoris is an ideal detection target for predicting the occurrence of ICUAW (13). This study aimed to verify the correlation between rectus femoris diameter change (ΔRF-d) and global muscle strength, so as to provide a non-invasive, bedside and cooperation-independent diagnostic tool for ICUAW.

## 2 Materials and methods

### 2.1 Inclusion and exclusion criteria

This prospective observational study was conducted in the 41-bed ICU of Army Medical Center (Chongqing, China) from March 2019 to March 2020. All patients admitted to the ICU were screened. Inclusion criteria: adult patients receiving invasive mechanical ventilation for no less than 5 days with endotracheal intubation initiated within 24 h before or after ICU admission.

Exclusion criteria: impaired consciousness with impaired communication, fewer than two limbs available for examination, pre-existing neurological or muscular disorders, or baseline functional weakness. Baseline daily living capacity was retrospectively collected via medical records and family interviews within 24 h of admission using ADL criteria. Patients who required assistance for routine self-care, suffered from stroke sequelae, advanced osteoarthritis, severe chronic heart or lung disease with long-term bedridden state were excluded to eliminate pre-existing dysfunction.

All assessments were performed only when patients reached Richmond Agitation-Sedation Scale (RASS) score of 0 (alert, calm, able to follow limb commands). Assessments were delayed and rechecked daily for patients with RASS < 0 (sedated) or RASS > 0 (agitated). The four-limb MRC strength score was independently measured by one ICU physician with over 5 years of experience following standard protocols. Rectus femoris ultrasonography with electrical stimulation was performed immediately after MRC scoring. Demographic and clinical data including age, gender, comorbidities, sepsis, ARDS, corticosteroid and neuromuscular blocker exposure, blood glucose, APACHE II and SOFA scores were recorded.

### 2.2 Rectus femoris contraction and muscle mass measurement

Attending physicians evaluated patients’ clinical status daily. Once patients regained clear consciousness and could cooperate with MRC assessment, the designated sonographer completed ultrasound measurements within 24 h. The timing of data collection varied individually based on each patient’s cooperative ability.

**FIGURE 1:**
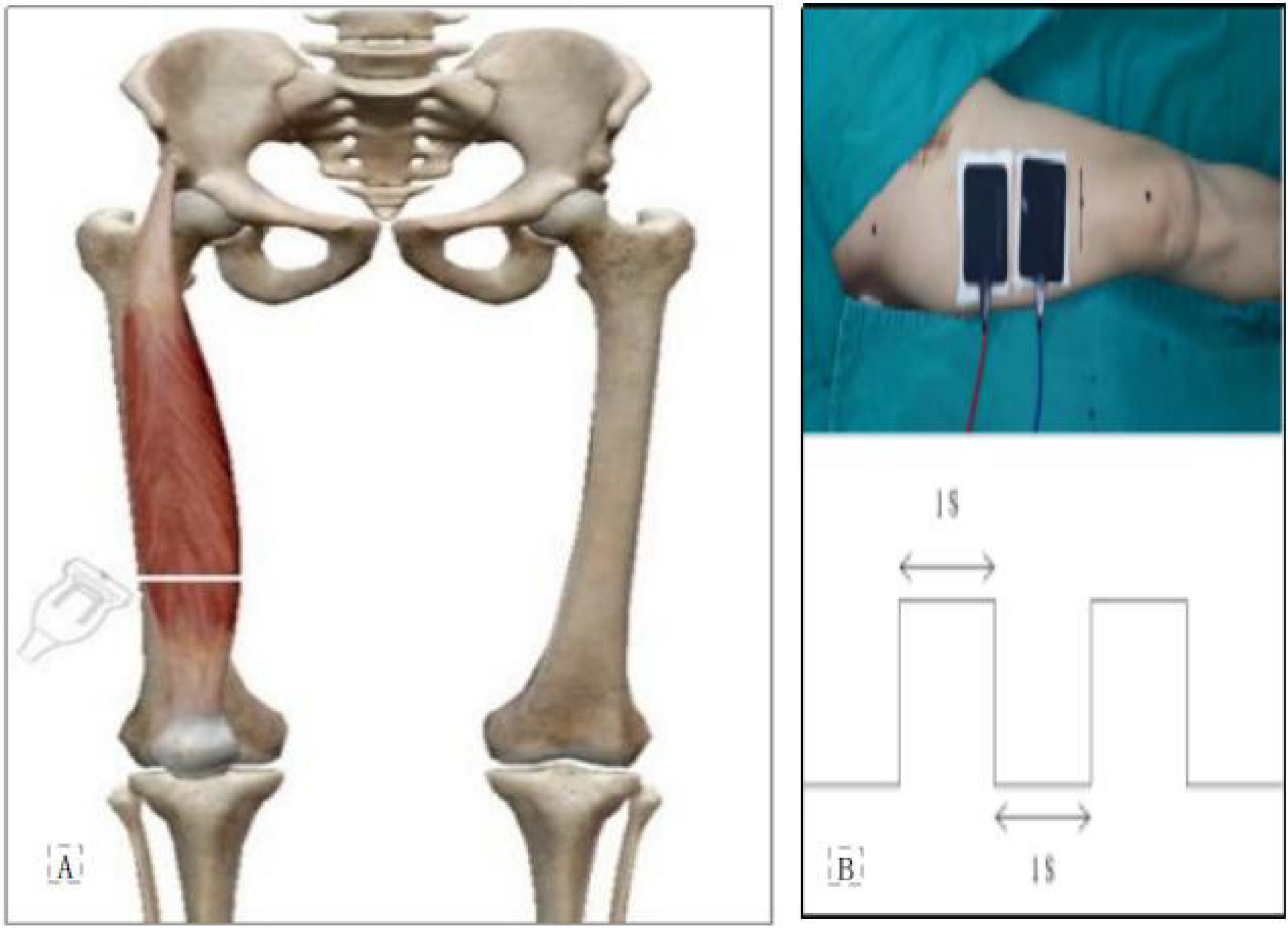
Schematic diagram of the measurement method. **(A)** Ultrasound acquisition points. **(B)** Electrode placement and electrical stimulation mode.

Patients were placed in the supine position with thighs fully extended. The ultrasound scanning site was marked at the distal quarter of the line connecting the anterior inferior iliac spine and the midpoint of the superior patellar border (Figure 1A) (15). Two 4 × 6 cm bipolar surface electrode patches were applied with their lower edges 1 cm above the scanning site. Electrical stimulation was delivered via a basic Fitzmann low-frequency therapeutic apparatus within clinically safe parameters: unidirectional square waves, pulse width 500 μs, frequency 50 Hz, and constant current of 30 mA (16, 17). Each stimulation burst lasted 1 second (ON 1 s), followed by a 1-second resting interval (OFF 1 s) (Figure 1B).

**FIGURE 2:**
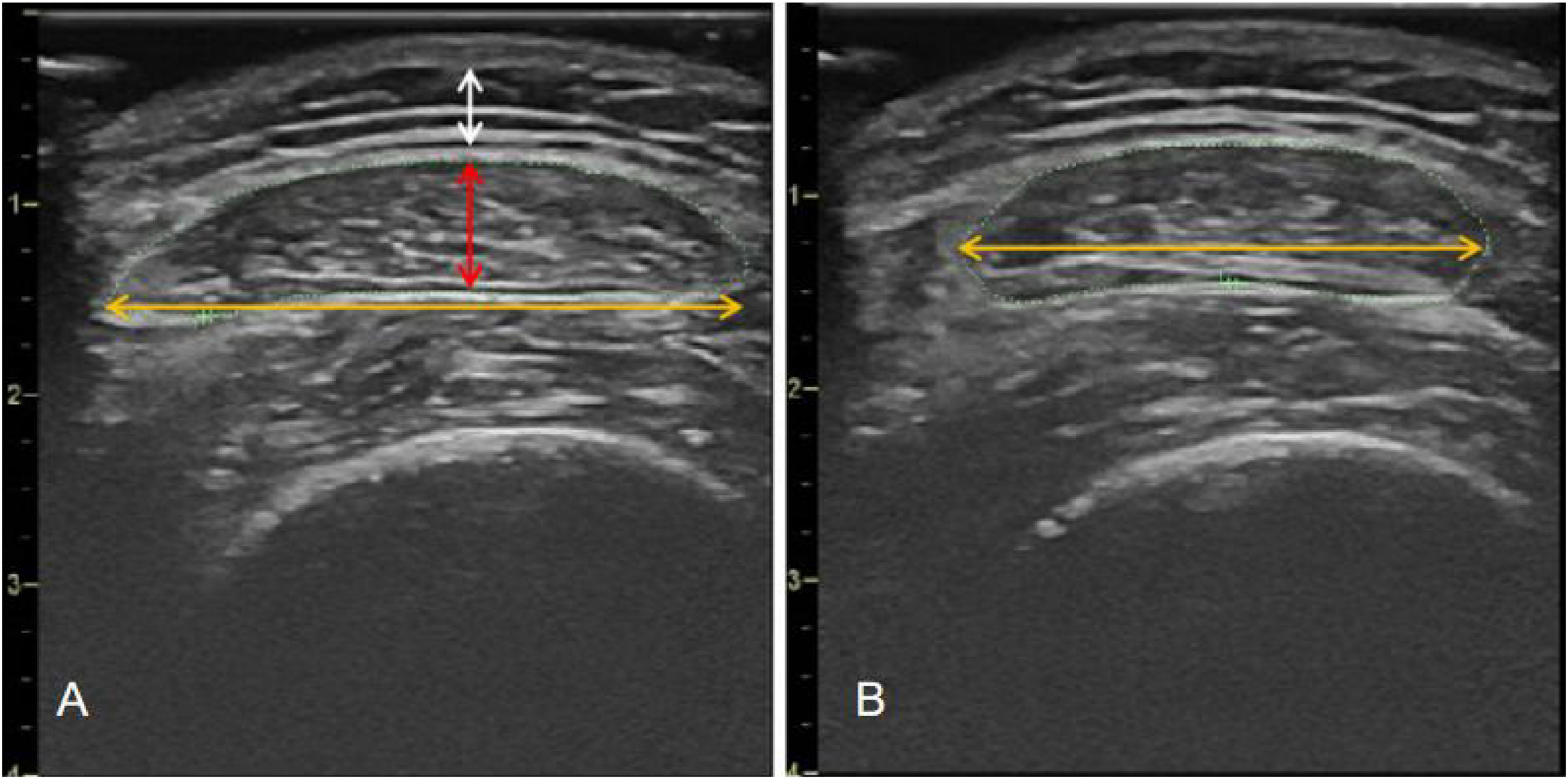
Ultrasonic measurement of rectus femoris transverse diameter for calculating ΔRF-d (rectus femoris rest-contraction transverse diameter difference, resting diameter minus contraction diameter). **(A)** Resting rectus femoris; yellow double-headed arrow = resting maximum transverse diameter; red double-headed arrow = resting muscle thickness; green outline = resting cross-sectional area. **(B)** Rectus femoris under neuromuscular electrical stimulation contraction; yellow double-headed arrow = contracted maximum transverse diameter (shorter than resting diameter). ΔRF-d equals the difference between resting transverse diameter and contracted transverse diameter; a larger ΔRF-d represents stronger muscle contractile function.

A GE Healthcare Vivid T8 color Doppler ultrasound system with an L6-12-RS linear probe was used. Adequate ultrasound gel was applied to obtain clear images without tissue compression. The probe was positioned perpendicular to the rectus femoris to capture transverse cross-sectional views. The diameter, thickness and cross-sectional area (CSA) of the rectus femoris were measured at rest and during maximal contraction (Figures 2A,B).

### 2.3 Rehabilitation therapy

A unified standardized rehabilitation protocol was adopted in our ICU, and standardized physical therapy was provided for all patients meeting the rehabilitation initiation criteria.

(1) Safety criteria for rehabilitation initiation: Relatively stable hemodynamics with norepinephrine or epinephrine infusion rate < 0.5 μg/kg⋅min; absence of active bleeding or severe arrhythmia; ventilator settings meeting safe mobilization thresholds; Richmond Agitation-Sedation Scale (RASS) ≤ 2.

(2) Stratified rehabilitation protocol: Stepwise training was delivered according to patients’ consciousness and muscle strength, including passive limb mobilization in bed, active joint movement, bedside sitting, bedside standing and ambulation out of bed. Neuromuscular electrical stimulation was combined for passive rehabilitation in debilitated patients unable to cooperate voluntarily.

(3) Standardized quality control: All rehabilitation procedures were performed by full-time professional physiotherapists following the unified early mobilization workflow of the department. Uniform standards were applied to the frequency, single-session duration and intensity of training, and the general rehabilitation framework was consistent across all groups.

#### 2.3.1 Calculated indicators

ΔRF-d = Resting maximum transverse diameter of rectus femoris − Maximum transverse diameter during maximal electrical stimulation contraction (Figure 2).

ΔRF-d is defined as the rest-to-contraction transverse diameter difference of rectus femoris (rest value minus contraction value). A larger ΔRF-d indicates better muscle contractile capacity.

ΔRF-CSA = Cross-sectional area at rest − Cross-sectional area at maximal contraction

Rectus femoris thickness = Maximum anteroposterior diameter at rest cross-section

Rectus femoris cross-sectional area = Cross-sectional area at rest

### 2.4 Nutritional therapy

Nutritional support was initiated once patients achieved hemodynamic stability with blood lactate < 2.5 mmol/L. For enteral nutrition (EN) initiation, additional abdominal and circulatory safety criteria must be satisfied: no active gastrointestinal bleeding, no intra-abdominal hypertension, and no severe abdominal distension or diarrhea. In patients with gastrointestinal perforation who received fistula feeding tubes, EN could be cautiously initiated after physician assessment, with norepinephrine or epinephrine infusion rate controlled below 0.5 μg/kg⋅min.

All patients received standardized nutritional management in accordance with critical care nutrition guidelines. Nutritional risk screening was performed within 24 h of ICU admission using the NRS-2002 scale. EN was started as early as possible for eligible patients; parenteral nutrition was supplemented when enteral intake failed to meet energy targets. The daily total energy target was set at 30 kcal/kg, with a carbohydrate-to-fat energy ratio of 6:4 and protein provision of 1.5 g/kg per day. Serum albumin was maintained within the range of 30–35 g/L. Data including timing of nutrition initiation, days required to reach energy targets, and total albumin transfusion volume within the first 7 ICU days were recorded for all subjects.

### 2.5 Objectivity of the study

All ultrasonic scans were performed by a senior attending intensivist with rich clinical experience, who remained blinded to patients’ clinical information. MRC muscle strength assessment was finished separately by a senior rehabilitation physician. Every ultrasonic parameter was measured in triplicate, and the mean value was used for statistical analysis. All statistical computations were completed independently by another associate chief physician of the ICU.

### 2.6 Ethics and registration

This study was approved by the Ethics Committee of the Army Characteristic Medical Center (Approval No. Yiyanlunshen 2019 No.17) and registered in the Chinese Clinical Trial Registry (ChiCTR2000028917). Informed consent was obtained in writing from all patients or their family members.

### 2.7 Statistical analysis

Continuous variables with a normal distribution were expressed as mean ± standard deviation, and comparisons between groups were performed using the independent-samples *t*-test. Non-normally distributed continuous variables were presented as median (25th, 75th percentiles) and analyzed with the Mann-Whitney U test. Categorical variables were described as n (%) and compared using the Chi-square (χ^2^) test. Multiple linear regression analysis was conducted to identify factors influencing the MRC muscle strength score, and receiver operating characteristic (ROC) curve analysis was used to evaluate the diagnostic efficacy of relevant indicators. A two-tailed *P* < 0.05 was considered statistically significant. For binary predictor variable (corticosteroid exposure), we assigned 1 = patients received corticosteroids, 0 = patients without corticosteroid use. To avoid selection bias caused by single-factor pre-screening, we adopted pre-defined hierarchical backward stepwise multiple linear regression without preliminary univariate filtering. A total of 12 clinical covariates were divided into three clinically logical blocks (4 variables per block) and entered sequentially into the model: Block 1 (demographic and illness severity: age, mean blood glucose, APACHE II, SOFA); Block 2 (infection, nutrition and rehabilitation: sepsis, NRS-2002, rehabilitation initiation time, corticosteroid use); Block 3 (rest and dynamic rectus femoris ultrasound indicators: resting thickness, resting CSA, ΔRF-CSA, ΔRF-d). The variable elimination threshold within each block was predefined as *P* > 0.10. Variance inflation factor (VIF) was calculated to test multicollinearity; VIF < 3 indicated no obvious collinearity. All statistical tests were two-tailed, with *P* < 0.05 defined as statistically significant.

## 3 Results

### 3.1 Patient enrollment

**FIGURE 3:**
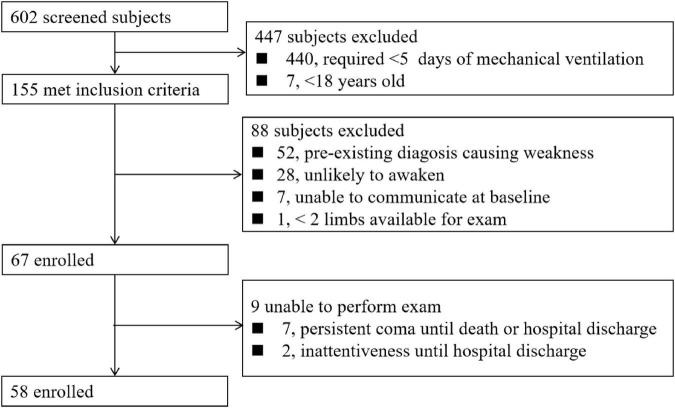
Flow chart of patient enrollment and exclusion.

A total of 602 patients were screened, with 58 finally enrolled in the study, including 32 cases (55.2%) in the ICUAW group and 26 cases (44.8%) in the NICUAW group (Figure 3).

### 3.2 Baseline characteristics

All enrolled patients maintained full independent self-care ability without chronic functional disability prior to ICU admission. Compared with the NICUAW group, patients in the ICUAW group were significantly older, and exhibited higher incidences of sepsis and corticosteroid exposure, as well as higher blood glucose levels (9.5 ± 1.2 vs. 8.7 ± 1.1, *P* < 0.05), APACHE II scores (21.1 ± 4.9 vs. 14.2 ± 4.7, *P* < 0.05), SOFA scores [11 (10.0, 14.0) vs. 8 (6.8, 10.3), *P* < 0.05] and NRS-2002 scores [6.5 (6.0, 7.0) vs. 5.0 (5.0, 6.0), *P* < 0.05]. In addition, the time to initiation of rehabilitation was markedly delayed in the ICUAW group [3.0 (2.3, 4.0) vs. 2.0 (1.0, 3.0), *P* < 0.05]. No significant intergroup differences were observed in gender, comorbidities, ARDS incidence, doses of neuromuscular blocking agents, timing of enteral/parenteral nutrition initiation, days to reach nutritional energy targets, or the proportion of patients receiving rehabilitation (Table 1).

**TABLE 1:** Comparison of demographic characteristics between the two groups.

Characteristic	Total	ICUAW	NICUAW	*P-*value
Subjects, %	58.0	32.0(55.2)	26.0(44.8)	
Age, years, median (IQR)	57.0(45.8, 72.3)	70.0(53.5, 75.0)	47.0(35.0, 56.5)	< 0.001
Sex, female (%)	27.0(46.6)	18.0(56.3)	9.0(34.6)	0.10
Co-morbidity				
Hypertension (%)	20.0(34.5)	13.0(40.6)	7.0(26.9)	0.28
Diabetes mellitus (%)	11.0(19)	6.0(18.8)	5.0(19.2)	1.00
Lung disease (%)	5.0(8.6)	4.0(12.5)	1.0(3.8)	0.49
Heart disease (%)	8.0(13.8)	7.0(21.9)	1.0(3.8)	0.11
Chronic kidney disease (%)	3.0(5.2)	2.0(6.3)	1.0(3.8)	1.00
Digestive disease (%)	4.0(6.9)	2.0(6.3)	2.0(7.7)	1.00
Hypercholesterolemia (%)	2.0(3.4)	1.0(3.1)	1.0(3.8)	1.00
Lacunar cerebral infarction (%)	4.0(6.9)	3.0(9.4)	1.0(3.8)	0.76
Admitting conditions				
Severe sepsis (%)	29.0(50.0)	23.0(71.9)	6.0(23.1)	< 0.05
ARDS (%)	51.0(87.9)	31.0(96.9)	20.0(76.9)	0.06
Any corticosteroid use (%)	24.0(41.4)	21.0(65.6)	3.0(11.5)	< 0.05
Any neuromuscular blocker use (%)	1.0(1.7)	1.0(3.1)	0.0(0.0)	1.00
Blood glucose, mmol/L, mean ± SD	9.1 ± 1.2	9.5 ± 1.2	8.7 ± 1.1	< 0.05
APACHE II, mean ± SD	18.0 ± 5.9	21.1 ± 4.9	14.2 ± 4.7	< 0.05
Total SOFA score, median (IQR)	8.0(5.0, 10.0)	11.0(10.0, 14.0)	8.0(6.8, 10.3)	< 0.05
Nutritional status				
NRS-2002 score, median (IQR)	6.0(5.0, 7.0)	6.5(6.0, 7.0)	5.0(5.0, 6.0)	< 0.05
Baseline prealbumin (ICU admission), mg/L, median (IQR)	99.9(78.1, 122.9)	91.5(77.9, 112.6)	105.1(78.1, 151.4)	0.21
Nutritional therapy initiated (%)	56.0(96.6)	30.0(93.8)	26.0(100.0)	0.20
Nutrition initiation time, days, median (IQR)	1.0(1.0,1.0)	1.0(1.0,1.3)	1.0(1.0,3.8)	0.12
Enteral nutrition initiated (%)	52.0(89.7)	28.0(87.5)	24.0(92.3)	0.55
Enteral nutrition initiation time, days, mean ± SD	2.3 ± 1.8	2.5 ± 2.0	2.1 ± 1.62	0.45
Achieving EN 30 kcal/kg energy goal, days (%)	42.0(72.4)	21.0(65.6)	21.0(80.8)	0.20
Time to reach enteral nutrition target of 30 kcal/kg, days, mean ± SD	4.9 ± 6.5	6.4 ± 8.2	3.3 ± 4.0	0.13
Mean albumin infusion dose in first 7 days (target serum albumin > 30 g/L), g, mean ± SD	18.2 ± 12.5	20.5 ± 14.0	15.4 ± 10.0	0.12
Rehabilitation status				
Rehabilitation training (%)	52(89.7)	28(87.5)	24(92.3)	0.68
Timing of rehabilitation initiation, days, median (IQR)	3.0(2.0, 3.0)	3.0(2.3, 4.0)	2.0(1.0, 3.0)	< 0.05

### 3.3 Rectus femoris contraction and muscle mass

The timing of assessments varied because MRC testing required patients to be alert and cooperative. The duration of endotracheal intubation at the time of measurement was comparable between the ICUAW and non-ICUAW groups (*P* > 0.05). Patients in the ICUAW group were older and had higher APACHE II and SOFA scores, indicating more severe overall illness. Compared with the non-ICUAW group, the ICUAW group displayed significantly smaller resting rectus femoris cross-sectional area [1.1 (0.7, 1.2) vs. 1.4 (0.9, 2.0), *P* < 0.05], rectus femoris thickness [0.6 (0.4, 0.7) vs. 0.7 (0.6, 1.0), *P* < 0.05], Δrectus femoris cross-sectional area [0.0 (0.0, 0.1) vs. 0.1 (0.1, 0.3), *P* < 0.05] and ΔRF-d (rest-contraction transverse diameter difference, resting minus contracted diameter) [0.0 (0.0, 0.1) vs. 0.2 (0.1, 0.4), *P* < 0.05] (Table 2). No intergroup differences were found in thigh circumference or subcutaneous fat thickness. These findings suggest that patients with ICUAW suffered more severe rectus femoris atrophy and poorer contractile function alongside more critical systemic conditions.

**TABLE 2:** Rectus femoris ultrasound parameters.

	Total	ICUAW	NICUAW	*P-*Value
Rectus femoris muscle area, cm^2^, median (IQR)	1.1(0.8; 1.6)	1.1(0.7; 1.2)	1.4(0.9; 2.0)	< 0.05
Rectus femoris muscle thickness, cm, median (IQR)	0.7(0.5; 0.9)	0.6(0.4; 0.7)	0.7(0.6; 1.0)	< 0.05
RF-d, cm, mean ± SD	2.4 ± 0.6	2.5 ± 0.6	2.4 ± 0.6	0.87
Δ Area of rectus femoris, cm^2^, median (IQR)	0.1(0.0; 0.1)	0.0(0.0; 0.1)	0.1(0.1; 0.3)	0.00
Δ RF-d, cm, median (IQR)	0.1(0.0; 0.3)	0.0(0.0; 0.1)	0.2(0.1; 0.4)	0.00
Thigh circumference, cm, mean ± SD	38.3 ± 6.6	37.3 ± 8.0	39.5 ± 4.0	0.20
Thigh subcutaneous fat thickness, cm, mean ± SD	0.73 ± 0.3	0.74 ± 0.4	0.72 ± 0.3	0.79
Days of endotracheal intubation at measurement, days, median (IQR)	7.5(5.0,13.0)	8.0(5.0,17.0)	6.0(5.0,12.0)	0.24

### 3.4 Factors influencing the mean MRC score and diagnostic efficacy by ROC curve analysis

Hierarchical backward stepwise multiple linear regression was conducted to identify independent predictors of the mean MRC score. Twelve predefined clinical covariates (corticosteroid use, sepsis, SOFA score, rehabilitation initiation time, mean blood glucose, age, NRS-2002 score, APACHE II score, rectus femoris thickness, resting rectus femoris cross-sectional area, ΔRF-d, Δrectus femoris cross-sectional area) were divided into three blocks with four parameters per block and entered sequentially into the model; the variable removal threshold within each block was set at *P* > 0.10. After iterative removal of covariates that failed to meet this predefined threshold across all blocks, the final stable model retained two independent predictors: ΔRF-d (*P* < 0.001) and corticosteroid use (*P* < 0.001). All variance inflation factor (VIF) values were < 3.0, indicating no prominent multicollinearity between variables. After full adjustment for demographic characteristics, critical illness severity scores, nutritional risk, rehabilitation initiation timing, sepsis status and other rectus femoris ultrasonic measurements, only ΔRF-d and corticosteroid use were confirmed to exert significant independent effects on the mean MRC score (Table 3).

**TABLE 3:** Multiple linear regression analysis of factors influencing the mean MRC score.

Variable	*B*	B SE	β	*t*	*P*-value	VIF
Constant	3.283	0.248	–	13.240	< 0.001	–
Corticosteroid use	−1.219	0.291	−0.425	−4.191	< 0.001	1.247
ΔRF-d	3.487	0.796	0.444	4.382	< 0.001	1.247
*F*			33.10			
*P*			< 0.001			
Adjusted *R*^2^ = 0.53 Regression equation: Mean MRC = 3.28 + 3.49 × ΔRF-d − 1.22 × Corticosteroid use						

**FIGURE 4:**
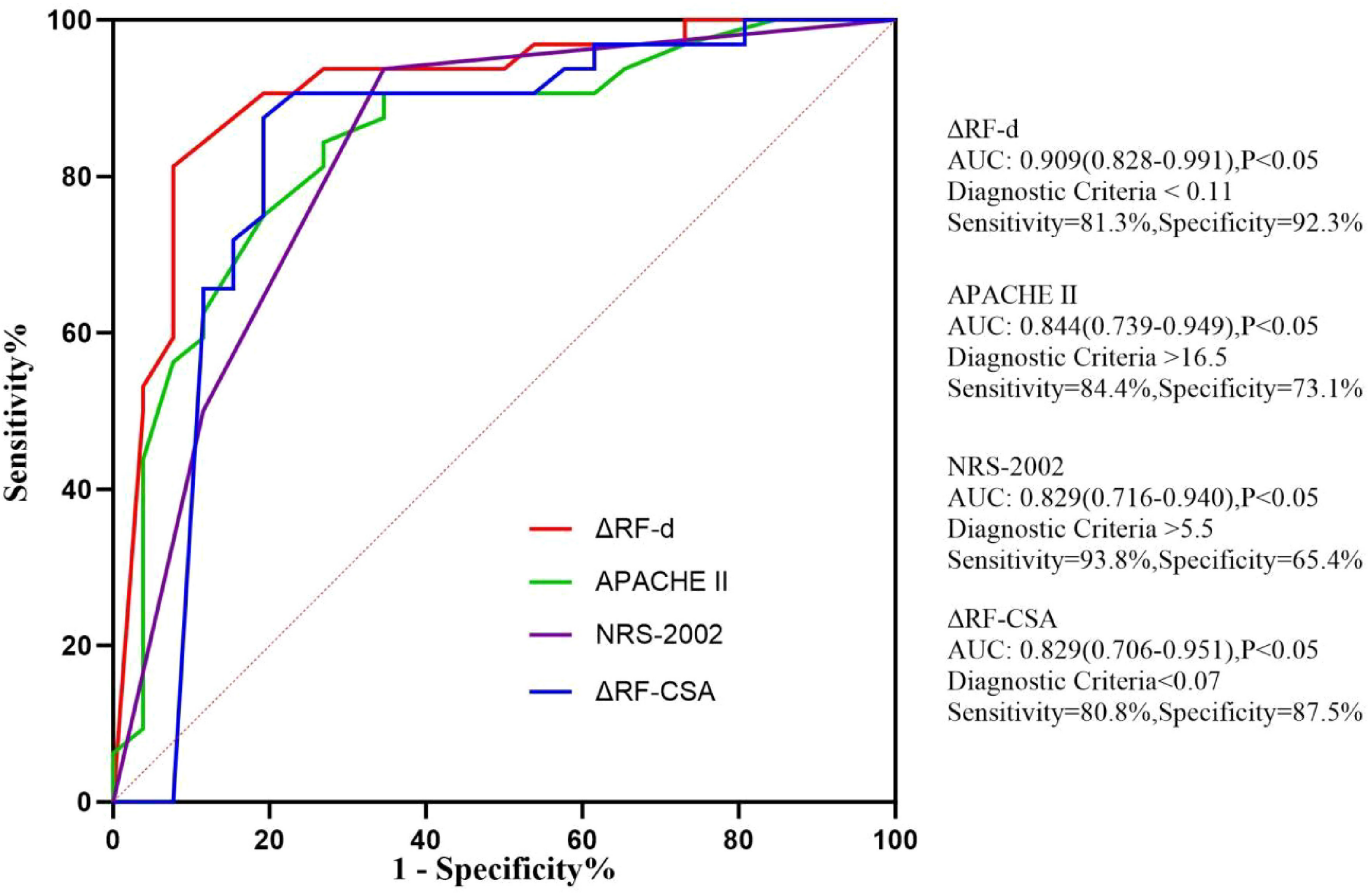
Diagnostic efficacy of ΔRF-d, APACHE II, NRS-2002, ΔRF-CSA for ICU-acquired weakness. ROC curves of four indicators with AUC > 0.8 for diagnosing ICUAW: ΔRF-d (AUC = 0.909), APACHE II score (AUC = 0.844), NRS-2002 score (AUC = 0.829), ΔRF-CSA (AUC = 0.829). The AUC values of age and corticosteroid exposure were below 0.8 and not presented here.

Receiver operating characteristic (ROC) curve analysis was applied to compare the diagnostic performance of corticosteroid use, sepsis, SOFA score, rehabilitation initiation time, mean blood glucose, age, NRS-2002 score, APACHE II score, rectus femoris thickness, resting rectus femoris cross-sectional area, ΔRF-d and Δrectus femoris cross-sectional area for ICUAW, with the MRC score as the diagnostic reference standard. The results revealed four indicators with an AUC greater than 0.8 and favorable diagnostic efficiency (Figure 4): ΔRF-d (AUC = 0.909, sensitivity = 81.3%, specificity = 92.9%, positive predictive value = 92.3%, negative predictive value = 81.2%), APACHE II score (AUC = 0.844, sensitivity = 84.4%, specificity = 73.1%, positive predictive value = 79.4%, negative predictive value = 79.1%), NRS-2002 score (AUC = 0.828, sensitivity = 93.8%, specificity = 65.4%, positive predictive value = 76.9%, negative predictive value = 89.5%) and ΔRF-CSA (AUC = 0.829, sensitivity = 80.8%, specificity = 87.5%, positive predictive value = 84.9%, negative predictive value = 84.0%).

## 4 Discussion

The results of multiple linear regression and ROC curve analyses in this study indicated that the electrical stimulation-induced rest-contraction transverse diameter difference of rectus femoris (ΔRF-d, resting diameter minus contraction diameter) can independently and accurately assess overall muscle strength in critically ill patients, with an AUC of 0.909 for the diagnosis of ICUAW. In terms of diagnostic discrimination, ΔRF-d yielded numerically higher performance than static morphological indicators including resting rectus femoris thickness and cross-sectional area; the latter two metrics merely reflect static muscle volume rather than functional muscle status.

ICU-acquired weakness (ICUAW) is a prevalent complication in the intensive care unit, affecting more than half of all critically ill ICU patients (18). Standard ICUAW diagnosis relies on cooperative awake patients via Medical Research Council (MRC) scale and handgrip dynamometry, yet the clinical detection rate remains low, as most patients stay sedated or comatose and cannot complete voluntary motor assessments during critical illness (14). Electrophysiological testing can differentiate primary neural and myopathic injuries in unconscious sedated patients with high sensitivity and specificity, but its wide clinical application is limited by sophisticated equipment and technical expertise (19). Muscle echo intensity analysis requires specialized software and professional training, while muscle biopsy is invasive and rarely adopted as routine monitoring by clinicians and patients (20, 21). A nationwide cross-sectional survey in China revealed major deficiencies in ICUAW assessment practice among domestic critical care staff: only 27% of respondents had managed ICUAW patients, 53% relied on subjective clinical experience for evaluation, merely 12% used standardized ICUAW assessment tools, and 35% consulted physiotherapists for judgment (20). These findings demonstrate a general lack of standardized, systematic ICUAW evaluation strategies. Developing a convenient, easy-to-perform assessment technique to overcome existing limitations is therefore critical. In the present study, ultrasound and neuromuscular electrical stimulation devices are universally available in ICUs. Our assessment protocol is non-invasive, radiation-free, safe, patient-cooperation-independent, easy to implement at bedside, time-saving, repeatable, cost-effective, and does not require specialized analytical software; clinical staff can master the procedure after brief training.

ICUAW is a severe, highly prevalent complication among critically ill patients with intricate pathophysiological regulatory networks (22). Its core manifestations include marked skeletal muscle atrophy and progressive limb weakness, which prolong mechanical ventilation duration and ICU length of stay, raise in-hospital and long-term all-cause mortality, and permanently impair patients’ long-term mobility and health-related quality of life (23). The pathogenesis of ICUAW is multifactorial, involving dysfunction of the neuromuscular unit, suppressed muscle protein synthesis, and accelerated protein breakdown (14). Prolonged immobilization in the ICU rapidly inhibits anabolic signaling pathways such as the AKT/mTOR cascade while activating proteolytic pathways (24). Microcirculatory dysfunction also contributes to ICUAW development by disrupting muscular oxygen delivery and utilization (25). The ubiquitin-proteasome system serves as a core mediator of intracellular protein degradation, and its aberrant activation is recognized as one of the central molecular mechanisms underlying ICUAW (26). The synergistic effects of these pathways jointly drive severe muscle mass loss and progressive weakness.

ICUAW is characterized by preferential myosin depletion, and myosin acts as the molecular motor for muscle contraction (14). Since ΔRF-d directly mirrors contractile capacity, this mechanism explains our finding that ΔRF-d independently predicts muscle strength, with markedly impaired rectus femoris contraction and overall weakness observed in ICUAW patients. Although ICUAW patients exhibit more severe muscle atrophy, the decline in muscle strength cannot be fully explained by reduced muscle volume alone, reflecting the dissociation between muscle mass and contractile function (27). Patients with chronic atrophy (e.g., chronic obstructive pulmonary disease, chronic kidney disease) may retain normal muscle strength (28), while individuals with preserved muscle mass (e.g., hypokalemic periodic paralysis) can present severe weakness (29), and critically ill patients face far more complex internal environmental disturbances. One previous study identified the rate of decrease in pennation angle as the optimal sonographic predictor of ICUAW (30). Another report reported favorable diagnostic accuracy for Δrectus femoris thickness and cross-sectional area, proposing that muscle mass changes better reflect muscle strength, with SOFA and APACHE II scores also demonstrating satisfactory predictive value for ICUAW (31). Consistent with prior literature, our study found that resting rectus femoris cross-sectional area exerted no independent effect on MRC scores. Although resting rectus femoris thickness correlated with muscle strength, SOFA and APACHE II scores failed to independently predict MRC values. Nevertheless, ROC comparison revealed that ΔRF-d, APACHE II, NRS-2002, ΔRF-CSA (AUC > 0.8) outperformed resting rectus femoris thickness (Figure 4). Resting rectus femoris thickness showed suboptimal diagnostic efficacy in our cohort, while another study reported that combined rectus femoris/vastus intermedius thickness measured on ICU admission plus SOFA score predicted subsequent ICUAW better than delayed muscle atrophy measurements. Discrepancies across studies may stem from small sample sizes, variable timing of sonographic acquisition, and greater measurement bias for cross-sectional area (requiring manual contour tracing) compared with linear measurements of diameter and thickness. Collectively, these data highlight inherent limitations of static muscle mass parameters for strength evaluation. To date, few publications have investigated whether ΔRF-d independently predicts muscle strength and incident ICUAW.

Baseline intergroup comparisons showed that the ICUAW group was significantly older, with higher prevalence of sepsis, greater corticosteroid exposure, elevated blood glucose, higher APACHE II, SOFA and NRS-2002 scores, and substantially delayed rehabilitation initiation time. No intergroup differences were detected in gender, comorbidities, ARDS incidence, neuromuscular blocking agent dosage, timing of enteral/parenteral nutrition, days to energy target achievement, or rehabilitation implementation rate (Table 1). These baseline disparities align with established ICUAW risk factors reported in previous research (14), and their pathophysiological mechanisms explain the elevated susceptibility to weakness in this cohort.

First, advanced age is accompanied by physiological myofiber loss and impaired myosin synthesis, reducing muscular functional reserve and rendering older patients more vulnerable to contractile dysfunction following severe inflammatory insult. Second, sepsis triggers a systemic inflammatory storm, where pro-inflammatory cytokines including TNF-α and IL-6 overactivate the ubiquitin-proteasome pathway and selectively degrade contractile myofilaments (32). Persistent hyperglycemia suppresses the mTOR anabolic pathway and exacerbates insulin resistance, further inhibiting muscle protein synthesis (33). Consistent with our regression results that corticosteroid exposure yielded a negative regression coefficient (B = −1.219) for MRC score, corticosteroid administration acts as an independent risk factor for reduced muscle strength and ICUAW occurrence after adjusting for all clinical and ultrasound covariates (34). The cumulative burden of these risk factors synergistically increases ICUAW risk. Additionally, delayed rehabilitation initiation in the ICUAW group results in prolonged immobilization; absent passive contractile stimulation fails to restrain proteolysis, creating dual injury from critical illness stress plus disuse and exacerbating weakness.

Notably, the proportion of patients receiving rehabilitation was comparable between groups, with only delayed onset observed in the ICUAW cohort. This indicates that standard rehabilitation alone cannot fully counteract critical illness-related muscle damage, largely due to the absence of an early, objective, cooperation-free monitoring tool. Clinicians cannot identify subclinical weakness during sedation when MRC testing is unfeasible, missing the optimal window for early intervention. Static thickness and cross-sectional area measurements only quantify muscle volume and are confounded by edema and age. In contrast, the electrical stimulation-derived ΔRF-d established herein independently reflects contractile function and retains robust diagnostic performance after adjustment for age, illness severity, corticosteroid exposure, nutritional risk and other confounders, enabling early identification of high-risk patients. The two groups were balanced in ARDS prevalence, neuromuscular blocking agent administration and timing of nutritional support, which excludes major confounding interference from these indicators when comparing muscle strength. Although advanced age, sepsis, high disease burden and delayed rehabilitation initiation manifested as baseline risk factors for ICUAW in intergroup comparison, only corticosteroid exposure and ΔRF-d exerted independent predictive effects on MRC muscle strength after multivariate adjustment. Furthermore, the dynamic rectus femoris contraction index ΔRF-d is less susceptible to baseline confounders, which can stably quantify global muscle strength and possesses outstanding clinical discriminative capacity.

Standardized neuromuscular electrical stimulation (NMES) rather than individualized stimulation regimens was adopted to enable quantitative intergroup comparisons of ΔRF-d across all enrolled patients. As summarized in the 2026 systematic review by Daum et al., which included 44 ICU trials covering 50 distinct NMES protocols, there is no globally unified standard for bedside muscle function assessment in critically ill patients, and published protocols exhibit remarkable heterogeneity: stimulation frequency ranges from 20 to 121 Hz, current intensity 2 heterogeneity: stimulati1,400 μs, and on-off cycles vary between 0.4 and 30 s (35, 36). Conventional NMES interventions can be split into two main categories: long-duration rehabilitation training protocols with 3habilitation training pron be splto mitigate disuse muscle atrophy (typically using 45fre Hz for sedated patients) (37), and high-current stimulation regimens developed solely to elicit complete knee extension in unconscious critically ill subjects (38). Distinct from these long-term rehabilitative interventions, our ultrasound-driven ΔRF-d measurement tool targets rapid ICU-acquired weakness (ICUAW) bedside screening instead of chronic muscle rehabilitation, so standardized stimulation settings were implemented to exclude confounding bias arising from inconsistent individual stimulus loads that alter muscle contraction magnitude.

Gradient parameter pre-experiments identified our final fixed stimulation regimen: 50 Hz frequency, 500 μs pulse width, 30 mA constant current, paired with a 1 s on/1 s alternating cycle; the entire ultrasound acquisition process takes only 6tant to capture 3ss takes only 6tant current, p-relaxation cycles. Current clinical evidence indicates that 30s a Hz represents the mainstream frequency band for quadriceps stimulation in ICU populations, and low-frequency pulsed current produces greater contractile force and superior anti-fatigue performance compared to kilohertz-range high-frequency alternating current (36). Despite this supportive background evidence, the exact parameter combination used in the present work has not been validated by dedicated clinical trials. All perceived advantages of this scheme—sufficient penetration of superficial soft tissue to produce stable local rectus femoris contraction with minimal discomfort in alert, cooperative patients, suppressed intramuscular lactic acid buildup, reduced muscle fatigue and limb displacement during scanning—remain theoretical physiological conjectures rather than empirically verified results. Most rehabilitation-focused NMES protocols reported in Daumis contractiopt cycle durations exceeding 3 s (36), and no prior research has confirmed the anti-fatigue effect of our 1 s short alternating cycle.

Although personalized NMES titration can maximize single-patient muscle contraction amplitude, custom adjustment of frequency and current generates unquantifiable intergroup bias and weakens the comparability of quantitative markers like ΔRF-d. This study does not draw the general conclusion that standardized stimulation outperforms individualized regimens for all ICU muscle assessment scenarios; high-quality head-to-head comparative trials between the two strategies are still scarce in existing literature. Individualized parameter calibration is still recognized as the optimal intervention for long-term rehabilitation training, and our unified fixed protocol is merely a study-specific methodological design to fulfill cross-cohort muscle function comparison demands. Recent NMES studies published between 2018 and 2026 (including a 75 Hz long-duration protocol exclusively applied to deeply sedated patients) only provide broad background consensus that ultrasound combined with electrical stimulation enables patient-independent muscle evaluation (36–40). However, their stimulus frequency, current, pulse width and cycle configurations differ drastically from our low-intensity, short-cycle testing scheme for awake mechanically ventilated patients, and their experimental outcomes cannot be directly extrapolated as direct validation evidence for our parameter set, only serving as contextual background for ICU NMES applications. Collectively, all potential functional merits of our fixed low-intensity 50 Hz short-cycle NMES protocol are purely a priori theoretical deductions lacking targeted preclinical or clinical verification, and further controlled trials are required to validate its reliability for quantitative rectus femoris contractile measurement. Notably, we did not conduct DeLong pairwise comparison of multiple ROC curves in this single-center small sample cohort, so the higher AUC value of ΔRF-d only reflects a numerical advantage without statistically significant differences compared with other indicators.

Study limitations: This study was conducted at a single center with a limited sample size. Despite these limitations, the present work carries meaningful clinical implications. First, the proposed technique eliminates the requirement for patient cooperation, features straightforward operation, relies on widely accessible equipment, and is readily generalizable for routine ICUAW risk screening by critical care staff. Second, although modifiable ICUAW risk factors (corticosteroids, hyperglycemia, malnutrition, neuromuscular blocking agents, early parenteral nutrition, vancomycin, aminoglycosides, vasoactive agents) and protective interventions (early mobilization, timely enteral nutrition) are well recognized, ICUAW remains difficult to prevent. This gap may be attributed to the lack of a safe, simple, effective, repeatable bedside modality to quantify muscular status and objectively reflect therapeutic responses in a timely manner. Our study establishes a feasible method for global muscle strength assessment. Future work will validate whether early ΔRF-d measurement within 48 hours of ICU admission predicts subsequent ICUAW onset. Baseline functional status prior to ICU admission was retrospectively collected via medical records and family interviews without prospective standardized functional scoring, introducing mild recall bias. Subsequent cohorts will adopt the Barthel Index to stratify pre-illness functional capacity and mitigate confounding. This study only documented rehabilitation implementation and initiation time without quantifying daily training duration or exercise intensity, precluding detailed analysis of dose-response relationships between rehabilitation volume and muscular function. Further research will stratify rehabilitation dosage to precisely characterize the association between mobilization interventions and ICUAW.

## 5 Conclusion

ΔRF-d can effectively reflect global muscle strength in critically ill patients. As a cooperation-independent, bedside-accessible, non-invasive and repeatable indicator, it exhibits good clinical application prospects for ICUAW screening.

## Data Availability

The original contributions presented in this study are included in this article/supplementary material, further inquiries can be directed to the corresponding author.
